# Czynniki Ryzyka Rozwoju Miażdżycy u Otyłych Dzieci w Wieku 6-12 Lat

**DOI:** 10.34763/devperiodmed.20172103.259265

**Published:** 2017-10-28

**Authors:** Alicja Karney, Hanna Brągoszewska, Leszek Soluch, Mariusz Ołtarzewski

**Affiliations:** 1 Oddział Hospitalizacji Jednego Dnia, Instytut Matki i Dziecka, Warszawa, Polska; 2 Zakład Diagnostyki Obrazowej, Instytut Matki i Dziecka, Warszawa, Polska; 3 Centralne Laboratorium, Instytut Matki i Dziecka, Warszawa, Polska; 4 Zakład Badań Przesiewowych, Instytut Matki i Dziecka, Warszawa, Polska

**Keywords:** miażdżyca, otyłość, dzieci, atherosclerosis, obesity, children

## Abstract

Otyłość u dzieci prowadzi do zaburzeń metabolicznych i zmian strukturalnych w naczyniach, które są przyczyną rozwoju chorób układu krążenia i cukrzycy typu 2.

**Celem:**

badania była ocena czynników ryzyka miażdżycy u dzieci otyłych badanych w Oddziale Hospitalizacji Jednego Dnia Instytutu Matki i Dziecka.

**Materiał i metody:**

Zbadano 75 dzieci w wieku 6-12 lat (36 chłopców,39 dziewczynek) z BMI >97 centyla, stanowiącą grupę badaną. Grupę kontrolną stanowiło:36 dzieci w wieku 5-10 lat (18 chłopców,18 dziewczynek) z BMI 75-90.

Analizowano dane z wywiadu dotyczące otyłości, chorób układu krążenia i zaburzeń lipidowych w rodzinie, u badanych dzieci analizowano BMI, obwód pasa, lipidogram, stężenie insuliny, grubość kompleksu błony wewnętrznej i środkowej tętnicy szyjnej (IMT) w obu grupach.

**Wyniki:**

U 82,6% otyłych dzieci z grupy badanej stwierdzono w rodzinie otyłość, a u 72% choroby układu krążenia i zaburzenia przemiany lipidowej, u dzieci z grupy kontrolnej: u 34,2% była otyłość w rodzinie a u 36,8% choroby układu krążenia i zaburzenia lipidowe. Średni obwód pasa u otyłych dzieci wynosił 72,7 cm, w grupie kontrolnej 59,9 cm (p<0,001). Średnie stężenie cholesterolu, LDL, trójglicerydów było wyższe w grupie badanej (p<0,001). Poziom insuliny u otyłych był prawie dwukrotnie wyższy niż w grupie kontrolnej (±8,47 SD; p<0,003). Średnia grubość IMT u otyłych dzieci wynosiła 0,36 mm ; ±0,059 SD (strona prawa) i 0,37 mm; ±0,033 SD (strona lewa). W grupie kontrolnej: 0,323 mm; ±0,087 SD (strona prawa), 0,32mm; ±0,082 SD (strona lewa), różnice między grupami były istotne statystycznie. Znaleziono dodatnią korelację pomiędzy obwodem pasa i poziomem insuliny(p<0,03). Wnioski: Otyłość i zaburzenia lipidowe częściej dotyczą dzieci obciążonych rodzinnie otyłością i chorobami układu krążenia. Zaburzenia lipidowe statystycznie częściej występują u dzieci otyłych. IMT jest znamiennie większa u dzieci otyłych w porównaniu z grupą kontrolną co sugeruje, że zmiany miażdżycowe struktury naczyń szyjnych mogą wystąpić u dzieci otyłych już we wczesnym dzieciństwie. Pozwala je wykryć nieinwazyjna ultrasonograficzna metoda oceny IMT. Dzieci z otyłością, szczególnie trzewną mają wyższy poziom insuliny, co może przyczynić się do rozwoju insulinooporności.

U rodziców brak jest dostatecznej wiedzy o skutkach otyłości i jego wpływu na występowanie miażdżycy i chorób układu krążenia.

## Abstract (en)

Obesity in children causes metabolic and structural changes in blood vessels which lead to the development of cardiovascular diseases and type 2 diabetes.

**Aim:**

The aim of the study was to assess the risk factors for atherosclerosis in obese children studied in the One-Day Hospitalization Department of the Institute of Mother and Child.

**Material and methods:**

The study included 75 children aged 6-12 years (36 boys, 39 girls) with a BMI>97^th^ percentile. The control group consisted of 36 children aged 5-10 years (18 boys, 18 girls) with a BMI of 75-90. Analysis was conducted of family history regarding obesity, CVD, dyslipidemia. The children’s examination consisted of: BMI, waist circumference, cholesterol, LDL, HDL, triglycerides, and insulin. Both groups had their IMT (left and right) examined with ultrasound.

**Results:**

In the study group 82.6% of the obese children had a positive family history of obesity and 72% of CVD and dyslipidemia. Regarding the children from the control group, 34.2% had a family history of obesity and 36.8 of CVD and dyslipidemia. The mean waist circumference in the obese children was 72.7 cm, while in the control group it was 59.9 (p<0.001). The mean levels of lipids were higher in obese children (p<0.001). The insulin level was almost twice as high as in the control group (±8.47 SD; p<0.003). The mean IMT in obese patients was 0.36 mm; ±0.059 SD (right side) and 0.37 mm; ±0.033 SD (left side), while in the control group it was 0.32 mm; ±0.087 SD (right side) and 0.32 mm, ±0.082 SD (left side). The differences between the two groups were statistically significant. A positive correlation between waist circumference and insulin level was found (p<0.003).

**Conclusions:**

Obesity and dyslipidemia are more common among children with familial obesity and CVD. Dyslipidemia is statistically more widespread among obese children. IMT is significantly higher in obese children compared with the control group, suggesting that changes in the structure of carotid atherosclerosis may occur in obese children in early childhood. This can be diagnosed using the non-invasive IMT method measured with ultrasound. Children with obesity, especially visceral, have higher levels of insulin, which may contribute to insulin resistance. Parents lack sufficient knowledge about the effects of childhood obesity and its effects on atherosclerosis and cardiovascular diseases.

## Wstęp

Nadwaga i otyłość stanowią narastający problem w medycynie rozwojowej na całym świecie. Według raportu opracowanego przez The International Obesity Task Force co piąte dziecko w Europie ma nadwagę lub jest otyłe [1]. Również w naszym kraju obserwuje się coraz więcej dzieci otyłych. Wg badań polskich nadmierna masa ciała występuje u 8,7% dzieci i młodzieży w wieku 6-17 lat, a 3,4% ma rozpoznawaną otyłość [2, 3, 4]. Według raportu HBSC opracowanego w 2014 r. przez J. Mazur w Instytucie Matki i Dziecka nadwaga i otyłość dotyczy 14,8 dzieci w wieku 11-15 lat, przy czym w grupie chłopców odsetek wynosi 19,2 %, w grupie dziewcząt - 10,4%. Odsetek młodzieży z otyłością waha się od 0,8% u dziewcząt 15-letnich do 4,2% u chłopców 11-letnich. Badacze HBSC analizowali tendencje zmian w częstości występowania nadwagi i otyłości u 11-15-latków w 25 krajach Europy, Kanadzie i USA w latach 2002-2010 [5]. Zaobserwowano powolny efekt plateau w narastaniu otyłości w porównaniu z badaniami opublikowanymi w 2010 r. Efekt ten dotyczył młodzieży z ponad połowy analizowanych krajów zachodnich. Jedynie w krajach Europy Środkowo-Wschodniej obserwowano w tym czasie zwiększanie się odsetka młodzieży z nadwagą i otyłością. W Polsce zwiększał się on średnio o mniej więcej 1 punkt procentowy rocznie u chłopców i dziewcząt (HBSC 2014) [5].

Otyłość jest jednym z najważniejszych czynników rozwoju miażdżycy, która jest procesem chorobowym, a jego istotą są zmiany zapalno-proliferacyjne prowadzące do uszkodzenia ściany tętnicy. Zmiany miażdżycowe mogą rozwijać się jednocześnie w różnych naczyniach. Podstawą ich rozwoju jest dysfunkcja śródbłonka naczyń [6, 7, 8]. Czynnikami uszkadzającymi śródbłonek jest stres oksydacyjny związany z nadciśnieniem tętniczym, cukrzycą i hipercholesterolemią, podwyższony poziom wolnych rodników, toksyny uwalniane u osób palących wyroby tytoniowe. Ostatnie badania wykazują, że czynnikami rozpoczynającymi proces aterogenezy są także infekcje bakteryjne i wirusowe, wywołane przez takie patogeny, jak: Helicobacter pylori, Chlamydia pneumoniae, wirusy Herpes, cytomegalowirus czy Mykobakterie. Bardzo dokładnie został poznany udział lipidów w rozwoju zmian miażdżycowych w tętnicach, które kumulują się w obrębie błony wewnętrznej naczyń i zwiększają przepuszczalność śródbłonka. Wynikiem tego procesu jest modyfikacja lipidów, migracja i proliferacja miocytów oraz komórek zapalnych (limocytów T i makrofagów), jak również produkcja prozapalnych cytokin [6, 9].

Miażdżyca, prowadząca do uszkodzenia i zwężenia naczyń krwionośnych należy do głównych zdrowotnych zagrożeń cywilizacyjnych współczesnego świata. Procesy te zaczynają się już we wczesnym dzieciństwie i mogą prowadzić do rozwoju chorób układu krążenia w wieku średnim i starszym [7, 9, 10, 11].

Postępy w dziedzinie diagnostyki obrazowej pozwalają na mierzenie i ocenę grubości śródbłonka naczyń, który pierwszy ulega procesom patologicznym w dużych naczyniach obwodowych oraz w nerkach.

W tętnicach możemy wyróżnić błonę wewnętrzną (*tunica intima*), błonę środkową (*tunica media*) oraz błonę zewnętrzną (*tunica adventiva*). Kompleksem intima-media nazywamy struktury ściany naczyniowej mierzone od linii granicznej przydanka–błona środkowa do linii granicznej błona wewnętrzna– światło naczynia. Pomiar grubości kompleksu błony wewnętrznej i środkowej tętnicy szyjnej wspólnej (IMT − intima-media thickness) pozwala na ocenę wczesnych zmian miażdżycowych stosując nieinwazyjną metodę ultrasonograficzną [12, 13, 14, 15].

## Cel

Ocena czynników ryzyka rozwoju miażdżycy u dzieci w wieku 6-12 lat zgłaszających się do Oddziału Hospitalizacji Jednego Dnia z powodu otyłości.

## Materiał i metody

W Oddziale Hospitalizacji Jednego Dnia Instytutu Matki i Dziecka są diagnozowane i leczone między innymi dzieci z otyłością prostą. Dzieci z problemem otyłości stanowią ok. 2-3% wszystkich pacjentów badanych w Oddziale w ciągu roku.

Badania przeprowadzono u 75 dzieci z otyłością (BMI >97 centyla), które stanowiły grupę badaną i 36 dzieci z prawidłową masą ciała (BMI 75-90 centyl), które stanowiły grupę kontrolną. Wśród dzieci otyłych było 36 chłopców i 39 dziewczynek, w grupie kontrolnej 18 chłopców i 18 dziewczynek.

Do wykonania badań zastosowano następujące metody:

- wywiad dotyczący występowania rodzinnych czynników ryzyka miażdżycy (otyłość, choroby układu krążenia, zaburzenia przemiany lipidowej),
- ocena wskaźnika BMI,
- pomiar obwodu pasa,
- badania biochemiczne: lipidogram, TSH , fT3, fT4, stężenie glukozy i insuliny we krwi na czczo,
- badanie usg szyi – pomiar grubości kompleksu błony wewnętrznej i środkowej ściany tętnicy szyjnej wspólnej (IMT).

Badania krwi były wykonywane przy pomocy aparatu Cobas-Integra, usg szyi za pomocą aparatu usg firmy Philips U22. Pomiaru kompleksu błony wewnętrznej i środkowej tętnicy szyjnej dokonywano na ścianie dolnej tętnicy szyjnej, w pozycji typowej, w projekcji podłużnej, przy użyciu głowicy 7,5 MHz. Wykonywano dwa pomiary ze strony prawej i lewej, oznaczano średnią z pomiarów. Poziomy oznaczanych wskaźników lipidogramu porównywano do norm podanych przez NCEP (National Cholesterol Education Program) zależnych od wieku dzieci (za punkt odniesienia przyjęto wartości prawidłowe) [16].

Wszystkie otyłe dzieci zostały skierowane na konsultacje dietetyczne.

Do analizy statystycznej zastosowano: test Kołmogorowa-Smirnowa, test U Manna-Whitney’a, χ^2^, do badania korelacji – współczynnik korelacji rho-Spearmana.

## Wyniki

Z danych z wywiadu wynika, że w grupie dzieci z otyłością (grupa badana) dodatni wywiad rodzinny w kierunku otyłości (rodzice, rodzeństwo, dziadkowie) dotyczył 82,6% dzieci. W grupie kontrolnej otyłość w rodzinie dotyczyła tylko 34,2% dzieci. Różnica jest istotna statystycznie: χ^2^(1)=26,53; p<0,001.

W grupie badanej 32% dzieci miało otyłych obydwoje rodziców; 17,3% dzieci miało tylko otyłą matkę, natomiast 16% dzieci miało otyłego tylko ojca.

W grupie badanej 72% miało obciążony wywiad rodzinny dotyczący również chorób układu krążenia i zaburzeń lipidowych, podczas gdy w grupie kontrolnej dotyczyło to 36,8% dzieci. Różnica między grupami jest istotna statystycznie: χ^2^(1)=13,00; p<0,001.W tabeli I przedstawiono sumarycznie dane z wywiadu.

Dzieci z grupy badanej miały średnio 7,97 lat, zaś z grupy kontrolnej – 8,02 roku. Grupy nie różniły się istotnie pod względem wieku: t(110)=-0,15; p>0,05.

Średnie BMI w grupie dzieci otyłych wynosiło − 24,3 (±3,58 SD), a w grupie kontrolnej z prawidłową masą ciała − 19,00 ( ±3,69 SD).

Średni obwód pasa w grupie badanej wynosił 72,7 cm (±9,05 SD), w grupie kontrolnej 59,9 cm (±4,19 SD), różnica była istotna statystycznie, t(103)=8,171;p<0,001.

Prawidłowe wartości składników lipidogramu wg NCEP dla dzieci z grupy kontrolnej, do których porównywano wyniki badań uzyskanych badanych przez nas dzieci były następujące: cholesterol całkowity <170 mg/dl, LDL<110 mg/dl, HDL>45 mg/dl, trójglicerydy (TG): do 9 r.ż. <75 mg/dl, 10-18 r.ż.<90 mg/dl.

Dzieci z otyłością miały znacząco wyższe poziomy cholesterolu całkowitego i jego frakcji LDL oraz trójglicerydów. Średnie stężenie cholesterolu w tej grupie wynosiło 167 mg/dl ( ±28,42 SD), a w grupie kontrolnej 124 mg/dl ( ±28,63 SD). Różnica była znacząca statystycznie, t(111)=9,12; p<0,001. Wartości cholesterolu całkowitego powyżej 170 mg/dl dotyczyły 47% dzieci z otyłością (z grupy badanej).

**Tabela I:** Dane z wywiadu dotyczące otyłości i chorób układu krążenia w rodzinie − grupa badana. Table I. Data from the interview on obesity and cardiovascular disease in the family − study group.

	Grupa badana *Study group*		Grupa kontrolna *Control group*		Wartość p *P-value*
	n	%	n	%	
Otyłość w rodzinie *Obesity in the family*	62	82,6	12	34,2	p<0,001
Choroby układu krążenia i zaburzenia lipidowe w rodzinie *Cardiovascular disease and dyslipidemia in the family*	54	72	13	36,8	p<0,001

**Tabela II:** Charakterystyka kliniczna dzieci z grupy badanej i kontrolnej oraz analiza statystyczna wyników. Table II. Clinical characteristic of the study and control group and statistical analysis of the obtained tests results.

	Grupa badana *Study group*		Grupa kontrolna *Control group*		Wartość p *P-value*
Liczba pacjentów *Patients, n*	n=75		n=36		
Wiek, średnia, ±, lata *Age*, *mean, ±*, *yrs*	7,97 ±1,69		8,02 ±1,59		p>0,05
Płeć *Gender*					
Chłopcy/dziewczynki, liczba (%) *Boys/Girls, n, (%)*	n=36 (48)	n=39 (52)	n=18 (50)	n=18(50)	
BMI, średnia, ± SD *BMI*, *mean, ±* *SD*	24,3±3,56		19,00±2,94		p<0,001
Średni obwód pasa, ±SD, cm *Medium waist circumference, ±SD, cm*	72,7±9,05		59,9±4,19		p<0,001
Cholesterol całk. średnia, ±SD, mg/dl *Total Cholesterol, mean, ±SD*	167±28,42		124±28,63		p<0,001
HDL, średnia, ± SD, mg/dl *HDL*, *mean, ±SD*, *mg/dl*	30±13,23		31±12,86		p<0,001
LDL, średnia, ±SD, mg/dl *LDL, mean, ±SD, mg/dl*	101±25,15		72±25,66		p<0,01
TG, średnia, ±, mg/dl *TG, mean, ±, mg/dl*	86±45,59		63±44,47		p < 0,01
Insulina,średnia, ± SD,mU/l *Insulin,mean, ±SD,mU/l*	10,27±8,47		5,88±5,83		p< 0,01
IMT, średnia, ±SD, mm *IMT, mean, ±SD, mm*	Strona prawa *Right side*	Strona lewa Left side	Strona prawa *Right side*	Strona lewa *Left side*	
	0,36±0,059	0,37±0,033	0,32±0,087	0,32±0,082	p<0,05 strona prawa (*right side*)/ /p<0,01 strona lewa (*left side)*

Średnie stężenie frakcji LDL cholesterolu w grupie badanej wynosiło 101 mg/ dl ( ± 25,5 SD), a w grupie kontrolnej 72 mg/ dl ( ± 25,66 SD), różnica była istotna statystycznie, t(110)=6,870;p<0.001. Wartości LDL>110 mg/ dl obserwowano u 33% dzieci otyłych.

Średnie stężenie trójglicerydów w grupie badanej było wyższe i wynosiło 86 mg/ dl ( ±45,59 SD) w porównaniu z grupą kontrolną, u której było na poziomie 63 mg/ dl ( ±44,47 SD), różnica była istotna statystycznie, t(100)-3,028; p=0,003. Stężenie trójglicerydów >75mg/dl dotyczyło 61% dzieci z otyłością.

W grupie badanej (z otyłością) niższe stężenie frakcji HDL cholesterolu (<45 mg/ dl) obserwowano u 21% dzieci otyłych.

Stężenie insuliny na czczo u dzieci z grupy badanej było prawie dwukrotnie wyższe i wynosiło 10,27 mU/ l, w grupie kontrolnej było równe 5,88 mU/l, t(109)=3,054; p<0,01. Wszystkie badane dzieci miały we krwi prawidłowy poziom hormonów tarczycy oraz poziom glukozy na czczo. Na podstawie badań obrazowych stwierdzono, że u dzieci otyłych średnia grubość kompleksu ściany tętnicy szyjnej wewnętrznej i środkowej po stronie prawej wynosiła 0,36 mm; ±0,059 (zakres: 0,22-0,55 mm), a po stronie lewej 0,37 mm; ±0,033 (zakres 0,20-0,63 mm). Nie była to różnica istotna statystycznie.

W grupie kontrolnej wartości te były następujące: po stronie prawej 0,32 mm; ±0,087 (zakres: 0,22-0,43 mm), po stronie lewej 0,32mm ; ±0,082 (zakres: 0,22-0,41 mm). Nie była to różnica istotna statystycznie. U dzieci otyłych, stanowiących grupę badaną, średnia grubość kompleksu ściany wewnętrznej i środkowej tętnicy szyjnej wspólnej była większa niż w grupie kontrolnej, szczególnie dotyczyło to tętnicy lewej, różnica była istotna statystycznie, t(111)=2,394,p <0,01 po stronie lewej, natomiast po stronie prawej - t(111)=2,609, p<0,05. Dane zebrano w tabeli II.

Analizowano również korelacje pomiędzy badanymi parametrami w grupie dzieci otyłych. Nie znaleziono istotnej korelacji pomiędzy BMI, stężeniem cholesterolu i jego frakcjami oraz poziomem trójglicerydów a grubością kompleksu ściany tętnicy szyjnej wewnętrznej i środkowej. Znaleziono dodatnią korelację pomiędzy poziomem insuliny na czczo a obwodem pasa u dzieci otyłych, im większy był obwód pasa tym wyższy był poziom insuliny, r=0,313, p<0,05.

Wszystkie badane dzieci otyłe zostały skierowane na konsultację dietetyczną, ale tylko 35/75 dzieci zgłosiło się na wizytę: pod stałą opieką poradni dietetycznej pozostało 14/35 dzieci, 11/35 nie zgłosiło się na kolejny wyznaczony termin wizyty, a 10/35 dzieci było tylko na jednej wizycie i nie zgłosiły się na kolejne. 29/75 dzieci z grupy badanej zgłosiło się na badanie kontrolne do Oddziału Hospitalizacji Jednego Dnia, 20/29 z nich zgłosiło się po roku od pierwszej wizyty, a jedynie 9/29 po 2 latach. Siedmioro z 29 dzieci było dwukrotnie na wizycie kontrolnej, po roku i po 2 latach.

Należy podkreślić, że tylko połowa otyłych dzieci skorzystała z zaplanowanych konsultacji dietetycznych i tylko 18% pozostało pod stałą opieką dietetyka. Na wizyty kontrolne do Oddziału zgłaszała się mała liczba pacjentów pomimo zaproszeń listownych lub telefonicznych.

## Dyskusja

Otyłość jest narastającym problemem u dzieci polskich. Najczęściej mamy do czynienia z otyłością prostą związaną z nadmierną podażą energii w diecie. Jedynie w wyjątkowych przypadkach jest to otyłość wtórna do innych chorób lub uwarunkowana genetycznie. Na rozwój otyłości pierwotnej (prostej) ma wpływ wiele czynników, ale przede wszystkim tryb życia, nawyki żywieniowe, mała aktywność <zyczna lub jej brak. Otyłość częściej dotyczy dzieci, których rodzice są otyli, co wykazano w badaniu własnym, jak i innych autorów [2, 17].

Dzieci otyłe częściej mają zaburzenia gospodarki lipidowej, szczególnie dotyczy to stężenia trójglicerydów, cholesterolu całkowitego i jego frakcji LDL. Według ostatnich doniesień Polskiego Towarzystwa Lipidologicznego, to właśnie frakcja LDL cholesterolu całkowitego ma największe znaczenie dla rozwoju blaszki miażdżycowej i nagłych zdarzeń sercowo-naczyniowych u dorosłych.

W badaniu własnym średnie stężenie cholesterolu i jego frakcji LDL oraz trójglicerydów było znamiennie wyższe u dzieci z otyłością, co jest zgodne z doniesieniami wielu innych autorów [11, 17, 18, 19].

Wiele danych wskazuje, że markery i czynniki ryzyka sercowo-naczyniowego wykrywane w dzieciństwie, często stwierdzane są również u tych samych osób w wieku dorosłym. Najczęściej mamy do czynienia z nadmierną masą ciała, zaburzeniami w zakresie gospodarki lipidowej czy węglowodanowej [17, 20, 21]. Występowanie zwiększonej masy ciała w młodym wieku może predysponować do rozwoju nadwagi i otyłości w okresie późniejszym. Około 50% otyłych nastolatków z BMI powyżej 95 centyla ma nieprawidłową masę ciała w wieku dorosłym [1, 2, 10].

Większość dzieci ze zdiagnozowanymi, ale nieleczonymi zaburzeniami lipidowymi będzie charakteryzowała się nimi także w późniejszym okresie życia. Obserwacje z *Bogalusa Heart Study* wykazały istotną korelację między czynnikami ryzyka rozwoju miażdżycy, takimi jak, nadciśnienie tętnicze i otyłość we wczesnym okresie życia, a zmianami miażdżycowymi w aorcie i w tętnicach wieńcowych w wieku dorosłym [20].

Przełomowe znaczenie dla zrozumienia patogenezy schorzeń układu krążenia miało dokonane ponad 20 lat temu odkrycie, że śródbłonek naczyniowy jest organem endo- i parakrynnym. Jego czynność odgrywa ważną rolę w regulacji napięcia ściany naczynia, proliferacji komórek mięśni gładkich, adhezji monocytów i leukocytów oraz procesów krzepnięcia, poprzez uwalnianie szeregu mediatorów rozszerzających naczynia utrzymuje właściwe napięcie ściany tętnicy. Nieprawidłowa funkcja śródbłonka traktowana jest jako wstępny etap rozwoju blaszki miażdżycowej [6, 22]. Celemajer opisał występowanie zaburzeń funkcji śródbłonka już u dzieci z rodzinną hipercholesterolemią i młodych dorosłych z czynnikami ryzyka choroby wieńcowej [13]. Dysfunkcja śródbłonka występuje na najwcześniejszych etapach miażdżycy, na długo przed wystąpieniem zwężeń widocznych w badaniach angiograficznych [9]. Monitorowanie czynników ryzyka w populacji dziecięcej jest istotne w prewencji rozwoju chorób sercowo-naczyniowych w późniejszym wieku. Pomiar grubości IMT jest jedną z tych metod. Ultrasonografia wysokiej rozdzielczości używana do pomiarów grubości IMT jest użyteczna w wykrywaniu wczesnych zmian w tętnicach szyjnych [7, 12, 13, 14]. Newman i wsp. na podstawie badań autopsyjnych ustalili, że od 5 do 10% dzieci w wieku 2-15 lat ma blaszki miażdżycowe w tętnicach wieńcowych [24]. Pomiar grubości IMT może być ważną metodą wykrywania zmian miażdżycowych u osób, które są obciążone rodzinnie chorobami układu sercowo- naczyniowego, ale jeszcze nie mają objawów [9, 25].

W badanej grupie dzieci w niniejszej pracy średnia grubość IMT u dzieci otyłych wynosiła: w lewej tętnicy 0,37 mm, w prawej 0,36 mm i była znamiennie statystycznie wyższa niż w grupie kontrolnej (0,32 mm). Podobne wyniki uzyskali inni badacze. W pracy Leite i wsp., oceniającej grubość IMT w grupie 150 portugalskich otyłych dzieci ( śr. wieku 12,9 lat), średnia grubość kompleksu u otyłych dzieci wynosiła – 0,472 mm, w grupie kontrolnej − 0,418 mm [26], w badaniach Lande i wsp. grubość IMT u otyłych dzieci była na poziomie 0,67 mm vs 0,63 mm w grupie kontrolnej [27], w badaniu polskim (Litwin i wsp.) średnia grubość IMT u otyłych dzieci z pierwotnym nadciśnieniem tętniczym wynosiła 0,67 mm, a w grupie kontrolnej 0,41 mm [28]. Elkiran i wsp. badając grupę 104 otyłych dzieci (śr. wieku 13 lat) również wykazali różnicę w grubości IMT u otyłych w porównaniu z grupą kontrolną (0,52 vs.0,36 mm) [18]. Badania Rumińskiej i wsp. w grupie 67 dzieci w wieku 7-15 lat, nie wykazały istotnych różnic w grubości kompleksu warstwy wewnętrznej i środkowej tętnicy szyjnej, co mogło wynikać ze zbyt małej grupy kontrolnej, jak sugerują sami autorzy, stwierdzono natomiast, że 91% dzieci otyłych miało IMT≥0,5 mm (0,57±0,08 mm) [11]. Należy jednak zauważyć, że grubość kompleksu w badaniu własnym jest mniejsza w porównaniu z wynikami innych autorów. Prawdopodobnie wynika to z wieku badanych dzieci. W naszej pracy dzieci były młodsze, średnia wieku ok.8 lat, w innych pracach powyżej 12 r.ż. Grubość IMT koresponduje ze zmianami histologicznymi i odzwierciedla obecność procesu miażdżycowego w naczyniach tętniczych. Wartości te zwiększają się z wiekiem, a także ściśle korelują z czynnikami ryzyka chorób sercowo-naczyniowych. U dorosłych wyższe wartości IMT stwierdzono u osób otyłych, z cukrzycą, nadciśnieniem tętniczym, palących papierosy. Poprzedzały one wystąpienie zawału mięśnia sercowego i udaru mózgu [14, 20]. Na wartość IMT w wieku dorosłym mają także wpływ czynniki ryzyka chorób układu krążenia występujące w wieku dziecięcym. W badaniach długofalowych wykazano, że do wyższych wartości IMT predysponuje nadmierna masa ciała dzieci i młodzieży, a zależność ta jest silniejsza w przypadku otyłości przetrwałej od dzieciństwa do wieku dorosłego [8, 10, 20, 29].

Analizowano również korelacje pomiędzy badanymi parametrami w grupie dzieci otyłych i nie znaleziono istotnej korelacji pomiędzy BMI, stężeniem cholesterolu i jego frakcjami oraz poziomem trójglicerydów a grubością kompleksu ściany tętnicy szyjnej wewnętrznej i środkowej (IMT). Według wielu autorów taka korelacja istnieje. W cytowanej wcześniej pracy Litwina i wsp. autorzy dowiedli, że w kształtowaniu wartości grubości IMT, oprócz biochemicznych czynników ryzyka rozwoju chorób sercowonaczyniowych rolę predyktora uszkodzenia ściany naczyń pełni także odpowiednio wysoka wartość BMI [28]. Do podobnych wniosków doszli Reinehr i wsp, w ich pracy wartość pomiaru IMT była istotnie powiązana z wielkością BMI [30]. W badaniu stwierdzono również dodatnią zależność między masą ciała (ale nie BMI) a grubością IMT. Podobne wyniki, jak w naszej pracy, dotyczące braku korelacji między profilem lipidowym a grubością IMT otrzymali Elkiran, Li, Rumińska [11, 18, 21]. Być może inne czynniki ryzyka chorób sercowo-naczyniowych odgrywają większą rolę aterogenną u dzieci i młodzieży, a działanie indukujące zmiany w naczyniach tętniczych ujawni się dopiero w wieku dorosłym. Wydaje się, że te różnice wiążą się z wiekiem dzieci. W naszej grupie badane były dzieci młodsze (6-12 lat), tymczasem we wszystkich cytowanych w piśmiennictwie pracach w badaniach brały udział dzieci powyżej 12 roku życia.

W badaniach własnych zwrócono uwagę na grupę dzieci, które zgłosiły się na badania kontrolne. Obserwowano następującą zależność: gdy BMI tych dzieci wzrastało to również wzrastała grubość IMT, natomiast w przypadku obniżenia się wartości BMI zmniejszała się grubość kompleksu. Z uwagi jednak na małą grupę dzieci, u których takie zależności obserwowano zjawisko to wymaga dalszych badaniach. Jest wysoce prawdopodobne, że obniżenie masy ciała u otyłych dzieci może zahamować, a nawet być może cofnąć rozpoczynający się proces miażdżycowy w naczyniach.

Insulina jest hormonem bezpośrednio działającym na komórki naczyń poprzez: zmniejszenie produkcji tlenku azotu, zwiększenie apoptozy komórek śródbłonka, obniżenie wychwytu glukozy, dodatkowo ma wpływ na ekspresję czynników wzrostowych i adhezyjnych oraz wzrost i migrację komórek mięśni gładkich naczyń. U osób z otyłością brzuszną dochodzi do rozwoju insulinooporności, która jest jednym z czynników rozwoju choroby niedokrwiennej serca. Rola hiperinsulinizmu w patogenezie miażdżycy jest złożona i nie do końca poznana [15, 21, 26, 30].

W badanej grupie dzieci stężenie insuliny na czczo było prawie dwukrotnie wyższe u dzieci otyłych niż w grupie kontrolnej (10,27 vs 5,88 umol/l). Znaleziono dodatnią korelację pomiędzy poziomem insuliny na czczo a obwodem pasa u dzieci otyłych, im większy był obwód pasa tym wyższy był poziom insuliny (r=0,313, p=0,01). Podobne obserwacje przedstawiają inni autorzy [11, 33]. Otyłość trzewna jest predyktorem insulinooporności, co w konsekwencji może prowadzić do rozwoju cukrzycy typu 2 w przyszłości.

Brak dostatecznej wiedzy wśród rodziców otyłych dzieci dotyczącej konsekwencji zdrowotnych związanych z nadmierną masą ciała wymaga podejmowania bardziej skutecznych działań uświadamiających społeczeństwo o skutkach nieprawidłowego stylu życia.

## Wnioski

1. Otyłość i zaburzenia lipidowe częściej dotyczą dzieci obciążonych rodzinnie otyłością i chorobami układu krążenia.
2. Zaburzenia lipidowe statystycznie częściej występują u dzieci otyłych niż u dzieci z prawidłową masą ciała.
3. Grubość kompleksu ściany tętnicy szyjnej wewnętrznej i środkowej jest znamiennie większa u dzieci otyłych w porównaniu z grupą kontrolna co sugeruje, że zmiany miażdżycowe struktury naczyń szyjnych mogą wystąpić u dzieci otyłych już we wczesnym dzieciństwie.
4. Wczesne zmiany miażdżycowe u dzieci pozwala wykryć nieinwazyjna ultrasonograficzna metoda oceny grubości kompleksu błon wewnętrznej i środkowej tętnicy szyjnej.
5. Dzieci z otyłością, szczególnie trzewną mają wyższy poziom insuliny co może przyczynić się do rozwoju insulinooporności.
6. Wczesne leczenie dzieci otyłych może zahamować proces rozwoju miażdżycy i zapobiec rozwojowi chorób układu krążenia u tych dzieci w przyszłości.
7. Brak dostatecznej wiedzy wśród rodziców otyłych dzieci dotyczącej konsekwencji zdrowotnych związanych z nadmierną masą ciała wymaga podejmowania bardziej skutecznych działań uświadamiających społeczeństwo o skutkach nieprawidłowego stylu życia prowadzącego do otyłości i jej powikłań.
